# Commentary: Role of Sterylglucosidase 1 (Sgl1) on the pathogenicity of *Cryptococcus neoformans*: potential applications for vaccine development

**DOI:** 10.3389/fmicb.2015.01112

**Published:** 2015-10-09

**Authors:** Luis R. Martinez

**Affiliations:** Department of Biomedical Sciences, New York Institute of Technology College of Osteopathic Medicine, New York Institute of TechnologyOld Westbury, NY, USA

**Keywords:** *Cryptococcus neoformans*, glycolipids, sterylglucosidase, T cells, vaccines

Cryptococcosis is an opportunistic fungal infection associated with AIDS. Differences in the clinical manifestations of cryptococcosis have been observed among encapsulated *Cryptococcus* species, with *neoformans* strains mostly isolated from individuals with impaired immunity (Dromer et al., 1994) and *gattii* strains from immunocompetent hosts (Speed and Dunt, 1995). This medically important fungus is globally distributed resulting in clinical disease in approximately 30% of HIV infected individuals mainly in areas such as South East Asia and sub-Saharan Africa (Powderly, 1993). Cryptococcossis is acquired after airborne fungi in the form of desiccated or poorly encapsulated yeast cells or as basidiospores enter the host by inhalation (Neilson et al., 1977; Levitz, 1991) resulting in asymptomatic disease or dormant infections. Progression toward pulmonary and systemic disease commonly occurs in individuals with severely compromised immune responses. There are 1 million cases of cryptococcal meningitis each year worldwide, resulting in approximately 600,000 deaths (Park et al., 2009).

*C. neoformans* infection presents formidable problems for the host immune response, including the presence of titan cells in tissue (Zaragoza and Nielsen, 2013), a lack of antibody responsiveness to capsular polysaccharide, and extensive accumulation of polysaccharide in tissue (Goldman et al., 1995). The presence of glucuronoxylomannan (GXM), the main capsular polysaccharide of *C. neoformans*, in tissue seems to be the most important contributor to the fungus pathogenesis given that the capsule is abundantly shed during infection and has been associated with immunosuppressive effects (Vecchiarelli, 2000), including an increase in HIV load (Pettoello-Mantovani et al., 1992) and cellular infectivity (Pettoello-Mantovani et al., 1994).

Despite its clinical relevance, there is no vaccine available for cryptococcosis. Several initiatives to generate artificial active immunity to prevent patients from developing this disease have been attempted including the development of a GXM bound to tetanus toxoid (Devi et al., 1991), a genetically engineered murine IFN-γ -producing cryptococcal strain (Wormley et al., 2007), and fungal components (Chaturvedi et al., 2014). Also, passive administration of antibodies has shown encouraging results in the laboratory (Casadevall et al., 1998). Nonetheless, although important insights have been acquired to better understand cryptococcosis, none of these initiatives have reached translational fruition. Thus, the development of alternative vaccination approaches is urgently needed.

Studies in mice with cell-mediated immunity deficiency support the importance of T-cells during cryptococcal infection given that these animals fail to mount T-dependent antibody responses (Graybill and Drutz, 1978; Cauley and Murphy, 1979). Conversely, thymus transplantation in these mice followed by fungal infection significantly prolongs survival (Graybill et al., 1979). Another piece of evidence supporting the role of T-cells in cryptococcal infection is that transference of T-cells from mice with cryptococcosis confers immunity to recipient mice (Lim and Murphy, 1980). In fact, the general consensus among medical mycologists is that it would be challenging to develop of an effective and protective anti-cryptococcal vaccine in individuals with impaired CD4^+^ T cell immunity, particularly in AIDS patients.

Rella et al., introduces a promising and innovative vaccination strategy to prevent cryptococcosis, by demonstrating that accumulation of sterylglucosides (SGs), a class of immunomodulatory glycolipids, in a genetically engineered non-pathogenic *C. neoformans* (Δ*sgl1*) strain that lacks the sterylglucosidase enzyme and administered to mice preceding fungal infection confers complete protection in animals challenged with lethal doses of either *C. neoformans* or *C. gattii* (Rella et al., 2015). Rodents immunized with the Δ*sgl1* strain a month prior to infection with wild-type cryptococci survived significantly longer than unexposed mice. Surprisingly, the Δ*sgl1* strain induces a CD4^+^ T cells independent immunity since previous immunization to infection of immunocompromised mice completely protected these animals against cryptococcosis. SGs immune stimulation suggests the possibility that prevention and sustained protection may be achievable in the context of T cell deficiency possibly by other immune mechanisms. For instance, CD8^+^ T cells may compensate for the loss of CD4^+^ T cells to facilitate protection against cryptococcosis in an IFN-γ-dependent manner (Lindell et al., 2005).

B cells play a critical role in protection against experimental cryptococcosis (Subramaniam et al., 2010; Szymczak et al., 2013). T cell independent immune responses result in the activation of B cells and the production of immunoglobulin M (IgM) which may be stimulated by SGs. For example, B-cell-deficient mice are susceptible to cryptococcal lung infection (Rivera et al., 2005), indicating an association between B cells and pulmonary inflammation control. In mice, IgM deficiency is linked with increased vulnerability to *C. neoformans* pulmonary infection and reduced phagocytosis, which can be restored by administration of IgM (Subramaniam et al., 2010). B cells from *C. neoformans* infected rodents produce fungal binding IgM and depletion of these cells resulted in impaired macrophage function facilitating cryptococcal dissemination to the brain (Rohatgi and Pirofski, 2012). Therefore, B cells might be SGs-glycolipid targets given that these humoral regulators enhance innate antifungal immunity via natural IgM, which promotes fungal containment in the lungs.

SGs have been shown to modulate cytokine production (Lee et al., 2007) suggesting their plausible implication in natural killer T (NKT) cell activation and macrophage polarization, innate immune responses that may be efficacious in prevention or treatment of cryptococcosis. NKT cells play an important role in the development of Th1 responses and host resistance to *C. neoformans*. For instance, NKT cells are increased in the lungs of mice infected with *C. neoformans*, associating the chemokine MCP-1 in their recruitment and accumulation (Kawakami et al., 2001a). Interestingly, glycolipid-activation of NKT cells increases IFN-γ levels and fungal clearance suggesting another possible target cell for stimulation by the Δ*sgl1* strain (Kawakami et al., 2001b). NKT cells also secrete IL-4, suggesting that this subset regulates both Th1- and Th2-polarization (Taniguchi et al., 2003). Likewise, the host response to *C. neoformans* in the lungs correlates with macrophage polarization (Davis et al., 2013), whereby M1 (classically activated) macrophages lead to Th1 responses (mainly IFN-γ dominated) and M2 (alternatively activated) macrophages lead to Th2 responses. M1 macrophages kill cryptococci more effectively than M2 macrophages suggesting that SGs-mediated changes in the cytokine environment can influence macrophage polarization, with M1 macrophages augmenting host defense against *C. neoformans*.

Further studies focusing on the immune responses generated by SGs and safety issues associated with delivering live-attenuated cryptococci, heat-killed fungi, or vesicle formulations containing SGs preparations are necessary. However, these findings are significant in the setting of HIV/AIDS immune deficiency suggesting that the Δ*sgl1* strain might provide a potential vaccination strategy against cryptococcosis. Rella et al., provide a proof of concept study that opens a novel area of research and a potential therapeutic strategy to prevent and reduce the devastating consequences of cryptococcosis.

## Conflict of interest statement

The author declares that the research was conducted in the absence of any commercial or financial relationships that could be construed as a potential conflict of interest.

## References

[B1] Casadevall A. Cleare W. Feldmesser M. Glatman-Freedman A. Goldman D. L. Kozel T. R.. (1998). Characterization of a murine monoclonal antibody to *Cryptococcus neoformans* polysaccharide that is a candidate for human therapeutic studies. Antimicrobial Agents Chemother. 42, 1437–1446. 9624491 10.1128/aac.42.6.1437 PMC105619

[B2] Cauley L. K. Murphy J. W. (1979). Response of congentitally athymic (nude) and phenotypically normal mice to *Cryptococcus neoformans* infection. Infect. Immun. 23, 644. 313367 10.1128/iai.23.3.644-651.1979 PMC414213

[B3] Chaturvedi A. K. Hameed R. S. Wozniak K. L. Hole C. R. Leopold Wager C. M. Weintraub S. T.. (2014). Vaccine-mediated immune responses to experimental pulmonary *Cryptococcus gattii* infection in mice. PLoS ONE 9:e104316. 10.1371/journal.pone.0104316 25119981 PMC4132117

[B4] Davis M. J. Tsang T. M. Qiu Y. Dayrit J. K. Freij J. B. Huffnagle G. B.. (2013). Macrophage M1/M2 polarization dynamically adapts to changes in cytokine microenvironments in *Cryptococcus neoformans* infection. MBio 4:e00264-13. 10.1128/mBio.00264-13 23781069 PMC3684832

[B5] Devi S. J. Schneerson R. Egan W. Ulrich T. J. Bryla D. Robbins J. B.. (1991). *Cryptococcus neoformans* serotype A glucuronoxylomannan-protein conjugate vaccines: synthesis, characterization, and immunogenicity. Infect. Immun. 59, 3700–3707. 1716613 10.1128/iai.59.10.3700-3707.1991 PMC258941

[B6] Dromer F. Varma A. Ronin O. Mathoulin S. Dupont B. (1994). Molecular typing of *Cryptococcus neoformans* serotype D clinical isolates. J. Clin. Microbiol. 32, 2364–2371. 7814467 10.1128/jcm.32.10.2364-2371.1994 PMC264067

[B7] Goldman D. L. Lee S. C. Casadevall A. (1995). Tissue localization of *Cryptococcus neoformans* glucuronoxylomannan in the presence and absence of specific antibody. Infect. Immun. 63, 3448–3453. 7642276 10.1128/iai.63.9.3448-3453.1995 PMC173475

[B8] Graybill J. R. Drutz D. J. (1978). Host defense in cryptococcosis. II. Cryptococcosis in the nude mouse. Cell. Immunol. 40, 263. 363279 10.1016/0008-8749(78)90334-9

[B9] Graybill J. R. Mitchell L. Drutz D. J. (1979). Host defense in cryptococcosis. III. Protection of nude mice by thymus transplantation. J. Infect. Dis. 140, 546–552. 10.1093/infdis/140.4.546 390064

[B10] Kawakami K. Kinjo Y. Uezu K. Yara S. Miyagi K. Koguchi Y.. (2001a). Monocyte chemoattractant protein-1-dependent increase of V alpha 14 NKT cells in lungs and their roles in Th1 response and host defense in cryptococcal infection. J. Immunol. 167, 6525–6532. 10.4049/jimmunol.167.11.6525 11714821

[B11] Kawakami K. Kinjo Y. Yara S. Koguchi Y. Uezu K. Nakayama T.. (2001b). Activation of Valpha14(+) natural killer T cells by alpha-galactosylceramide results in development of Th1 response and local host resistance in mice infected with *Cryptococcus neoformans*. Infect. Immun. 69, 213–220. 10.1128/IAI.69.1.213-220.2001 11119508 PMC97874

[B12] Lee J. H. Lee J. Y. Park J. H. Jung H. S. Kim J. S. Kang S. S.. (2007). Immunoregulatory activity by daucosterol, a beta-sitosterol glycoside, induces protective Th1 immune response against disseminated Candidiasis in mice. Vaccine 25, 3834–3840. 10.1016/j.vaccine.2007.01.108 17335944

[B13] Levitz S. M. (1991). The ecology of *Cryptococcus neoformans* and the epidemiology of cryptococcosis. Rev. Infect. Dis. 13, 1163. 10.1093/clinids/13.6.1163 1775849

[B14] Lim T. S. Murphy J. W. (1980). Transfer of immunity to cryptococcosis by T-enriched splenic lymphocytes from *Cryptococcus neoformans*-sensitized mice. Infect. Immun. 30, 5–11. 7002791 10.1128/iai.30.1.5-11.1980 PMC551268

[B15] Lindell D. M. Moore T. A. McDonald R. A. Toews G. B. Huffnagle G. B. (2005). Generation of antifungal effector CD8+ T cells in the absence of CD4+ T cells during *Cryptococcus neoformans* infection. J. Immunol. 174, 7920–7928. 10.4049/jimmunol.174.12.7920 15944298

[B16] Neilson J. B. Ivey M. H. Bulmer G. G. (1977). *Cryptococcus neoformans*: size range of infectious particles fro aerosolized soil. Infect. Immun. 17, 634. 332630 10.1128/iai.17.3.634-638.1977 PMC421174

[B17] Park B. J. Wannemuehler K. A. Marston B. J. Govender N. Pappas P. G. Chiller T. M. (2009). Estimation of the current global burden of cryptococcal meningitis among persons living with HIV/AIDS. AIDS 23, 525–530. 10.1097/QAD.0b013e328322ffac 19182676

[B18] Pettoello-Mantovani M. Casadevall A. Kollmann T. R. Rubinstein A. Goldstein H. (1992). Enhancement of HIV-1 infection by the capsular polysaccharide of *Cryptococcus neoformans*. Lancet 339, 21–23. 10.1016/0140-6736(92)90142-P 1370335

[B19] Pettoello-Mantovani M. Casadevall A. Smarnworawong P. Goldstein H. (1994). Enhancement of HIV type 1 infectivity *in vitro* by capsular polysaccharide of *Cryptococcus neoformans* and *Haemophilus influenzae*. AIDS Res. Hum. Retroviruses. 10, 1079–1087. 10.1089/aid.1994.10.1079 7826695

[B20] Powderly W. G. (1993). Cryptococcal meningitis and AIDS. Clin. Infect. Dis. 17, 837–842. 10.1093/clinids/17.5.837 8286622

[B21] Rella A. Mor V. Farnoud A. M. Singh A. Shamseddine A. A. Ivanova E.. (2015). Role of Sterylglucosidase 1 (Sgl1) on the pathogenicity of *Cryptococcus neoformans*: potential applications for vaccine development. Front. Microbiol. 6:836. 10.3389/fmicb.2015.00836 26322039 PMC4531891

[B22] Rivera J. Zaragoza O. Casadevall A. (2005). Antibody-mediated protection against *Cryptococcus neoformans* pulmonary infection is dependent on B cells. Infect. Immun. 73, 1141–1150. 10.1128/IAI.73.2.1141-1150.2005 15664957 PMC546959

[B23] Rohatgi S. Pirofski L. A. (2012). Molecular characterization of the early B cell response to pulmonary *Cryptococcus neoformans* infection. J. Immunol. 189, 5820–5830. 10.4049/jimmunol.1201514 23175699 PMC3518565

[B24] Speed B. Dunt D. (1995). Clinical and host differences between the two varieties of *Cryptococcus neoformans*. Clin. Infect. Dis. 21:28. 10.1093/clinids/21.1.28 7578756

[B25] Subramaniam K. S. Datta K. Quintero E. Manix C. Marks M. S. Pirofski L. A. (2010). The absence of serum IgM enhances the susceptibility of mice to pulmonary challenge with *Cryptococcus neoformans*. J. Immunol. 184, 5755–5767. 10.4049/jimmunol.0901638 20404271 PMC2885446

[B26] Szymczak W. A. Davis M. J. Lundy S. K. Dufaud C. Olszewski M. Pirofski L. A. (2013). X-linked immunodeficient mice exhibit enhanced susceptibility to *Cryptococcus neoformans* infection. MBio 4:00265-13. 10.1128/mBio.00265-13 23820392 PMC3705448

[B27] Taniguchi M. Harada M. Kojo S. Nakayama T. Wakao H. (2003). The regulatory role of Valpha14 NKT cells in innate and acquired immune response. Annu. Rev. Immunol. 21, 483–513. 10.1146/annurev.immunol.21.120601.141057 12543936

[B28] Vecchiarelli A. (2000). Immunoregulation by capsular components of *Cryptococcus neoformans*. Med. Mycol. 38, 407–417. 10.1080/mmy.38.6.407.417 11204878

[B29] Wormley F. L. Jr. Perfect J. R. Steele C. Cox G. M. (2007). Protection against cryptococcosis by using a murine gamma interferon-producing *Cryptococcus neoformans* strain. Infect. Immun. 75, 1453–1462. 10.1128/IAI.00274-06 17210668 PMC1828544

[B30] Zaragoza O. Nielsen K. (2013). Titan cells in *Cryptococcus neoformans*: cells with a giant impact. Curr. Opin. Microbiol. 16, 409–413. 10.1016/j.mib.2013.03.006 23588027 PMC3723695

